# Development of a rapid *comQ*-targeted quantitative polymerase chain reaction assay for specific identification and quantification of *Bacillus subtilis* subsp. *natto*

**DOI:** 10.1371/journal.pone.0355394

**Published:** 2026-08-03

**Authors:** Mayu Takizawa, Daisuke Kyoui, Rika Horiguchi, Ayano Sakurai, Taketo Kawarai, Naoki Narisawa

**Affiliations:** Department of Food Bioscience and Biotechnology, College of Bioresource Sciences, Nihon University, Fujisawa, Kanagawa, Japan; Tribhuvan University, NEPAL

## Abstract

*Bacillus subtilis* subsp. *natto* is a specialized functional group essential for fermented soybean production. Despite its industrial importance, genetic differentiation of this lineage remains challenging because of its high genomic similarity within the *B. subtilis* species complex. Here, we developed and validated a high-precision quantitative real-time polymerase chain reaction (qPCR) assay targeting the *comQ* gene—a core component of the quorum-sensing system. Comparative genomic analysis of 70 bacterial genomes, including 9 *B. subtilis* strains newly sequenced in this study, revealed that the *comQ* gene is under intense purifying selection (*dN/dS* = 0.10) and exhibits near-absolute conservation within the *natto* lineage (42 strains). While screening of these 70 genomes revealed a total of 15 atypical isolates (such as Miz-8 and PN1236) with reduced sequence identity (≤99%) or deletions in traditional metabolic (*bio* and *fliF*) and insertion sequence (IS) markers, their *comQ* loci remained 100% structurally intact. Phylogenetic analysis further confirmed that *comQ* forms a distinct monophyletic cluster for subsp. *natto*, providing superior diagnostic reliability over conventional markers, such as *bioF* or IS elements. By leveraging this highly conserved target, the established qPCR platform achieved an exceptionally wide, linear quantification framework (*R*^2^ = 0.9973, *E* = 94.2%) that completely bypasses peripheral genomic instability, maintaining high fidelity even under a 10,000-fold excess of non-target DNA. Direct validation on commercial fermented soybean matrices demonstrated a seamless correlation between molecular *comQ* copy number and viable cell count, underscoring its resilience against complex sample-derived enzymatic inhibition without the need for culture expansion. This assay transcends application in conventional industrial quality control—by providing a useful diagnostic and quantitative proxy, it provides a practical tool for tracking potentially opportunistic *natto* lineages linked to severe clinical bacteremia. Overall, we have established a reliable molecular surveillance method applicable to both industrial biotechnology and high-resolution clinical epidemiology.

## Introduction

*Bacillus subtilis* subsp. *natto* is an industrially and clinically relevant lineage within the *B. subtilis* species complex. Beyond its essential role in traditional soybean fermentation, this subspecies serves as a versatile biofactory for diverse bioactive compounds, including nattokinase, poly-γ-glutamic acid (γ-PGA), and vitamin K2 (MK-7), which offer multifaceted health benefits [1–4]. Recent studies have highlighted its broader functional repertoire, including antioxidant activities, angiotensin-converting enzyme-inhibitory effects for blood pressure regulation, and resilient probiotic properties that modulate the gastrointestinal health. Moreover, metabolites of this subspecies exhibit broad-spectrum antimicrobial [5], antibiofilm [6], and antiviral activities [7]. Although predominantly recognized for these industrial and health-promoting benefits, recent clinical data have also noted rare instances of opportunistic bacteremia associated with specific strains in clinical settings [8], underscoring the relevance of precise strain-level monitoring. Consequently, there is a growing need for a high-resolution tool capable of rapidly distinguishing these lineages to ensure both the quality and comprehensive safety in functional food applications.

Genetically, subsp. *natto* is nearly indistinguishable from other members of the species complex, such as the model strain *B. subtilis* subsp. *subtilis* 168, and cannot be resolved using standard phylogenetic markers like 16S rRNA, *gyrA*, or *recQ* [1,9–12]. Although multilocus sequence typing (MLST) provides higher resolution, it remains too labor-intensive and cost-inefficient for large-scale screening. Conventionally, molecular identification has relied on specific genetic signatures, including the biotin biosynthetic operon (*bioF* and *bioW*), flagellar structural genes (*fliF*), and various insertion sequence (IS) elements [9,13–15]. However, these approaches encounter significant technical and quantitative limitations: *bioF* and *fliF* require precise discrimination of small fragments; the single-nucleotide nonsense mutation in *bioW* cannot be differentiated via conventional PCR size-fractionation; and IS elements exhibit highly unstable copy number. Moreover, because these conventional markers are related to non-essential peripheral pathways, they are inherently prone to sporadic genomic rearrangements or deletions in atypical wild isolates, presenting a critical risk of false-negative diagnostic results [16].

To achieve rapid, one-step identification, we sought to exploit the unique evolutionary signatures embedded within the *comQXPA* quorum-sensing (QS) operon, which coordinates strain-specific social communication [15,17–22]. This locus encodes the signaling machinery consisting of the modifier ComQ, signaling peptide ComX, and sensor ComP. Because the *comQXP* module evolves as a tightly coupled functional unit to ensure signal-receptor fidelity, it undergoes intense purifying selection within the *natto* lineage, maintaining near-perfect sequence conservation to preserve QS integrity. Conversely, this co-evolutionary constraint drives extreme interlineage divergence from other subsp. *subtilis* populations that utilize different pherotypes. For instance, the ComQ protein in the *natto* lineage exhibits low amino acid sequence identity with that of the protein in the laboratory model strain 168, reflecting extreme interlineage variation driven by pherotype diversification [21]. This highly stable genetic signature—strong intralineage conservation coupled with profound interlineage variation—makes the *comQ* gene the most appropriate diagnostic window to establish a highly specific and reliable qPCR platform that is inherently more robust than the conventional markers.

In this study, we aimed to validate the potential of the *comQXPA* locus as a lineage-specific marker and to develop a robust diagnostic tool for *B. subtilis* subsp. *natto*. We performed a large-scale comparative genomic analysis of 70 bacterial genomes, including 9 *B. subtilis* strains newly sequenced in this study, to rigorously assess the intralineage conservation and inter-subspecies divergence of the operon. Leveraging these genomic insights, we targeted the *comQ* gene to design highly specific primers and established a quantitative real-time PCR (qPCR) assay. The specificity, sensitivity, and matrix tolerance of the assay were rigorously evaluated using a panel of diverse *Bacillus* species and various commercial *natto* products, providing a rapid, accurate, and high-throughput solution that effectively overcomes the technical limitations of the conventional molecular markers.

## Materials and methods

### Bacterial strains and culture conditions

A total of 37 strains were used to evaluate the specificity and sensitivity of the *comQ*-targeted assay (Table 1). They included 18 *Bacillus subtilis* subsp. *natto* strains, comprising commercial industrial starters (Miyagino and Naruse), type strains, three honey isolates (HK1−1, HK3−1, and HK11−1) previously characterized by Kimijima et al. [13], and five additional honey isolates (MH3−1, MH6−2, MH18−2, MH32−1, and MH33−2). For specificity testing, 19 non-target strains were included, focusing primarily on members of the closely related *B. subtilis* species complex. All strains were cultured overnight in Brain Heart Infusion (BHI) broth (Becton, Dickinson and Co., Franklin Lakes, NJ, USA) or Luria–Bertani (LB) broth at 37 °C, with shaking at 160 rpm, depending on their specific growth requirements.

**Table 1:** PCR-based identification of *Bacillus subtilis* subsp. *natto* and related *Bacillus* strains using various genetic markers.

Species	Strain	*comQ*	*bioF*	IS*4Bsu1*	IS*256Bsu1*	16S
*Bacillus subtilis* subsp. *natto*	NBRC 3013	+	+	+	+	+
*Bacillus subtilis* subsp. *natto*	Miyagino	+	+	+	+	+
*Bacillus subtilis* subsp. *natto*	Takahashi	+	+	+	+	+
*Bacillus subtilis* subsp. *natto*	Naruse	+	+	+	+	+
*Bacillus subtilis* subsp. *natto*	HK1−1	+	+	+	+	+
*Bacillus subtilis* subsp. *natto*	HK11−1	+	+	+	+	+
*Bacillus subtilis* subsp. *natto*	HK3−1	+	+	+	+	+
*Bacillus subtilis* subsp. *natto*	MH3−1	+	+	+	+	+
*Bacillus subtilis* subsp. *natto*	MH6−2	+	+	+	+	+
*Bacillus subtilis* subsp. *natto*	MH18−2	+	+	+	+	+
*Bacillus subtilis* subsp. *natto*	MH32−1	+	+	+	+	+
*Bacillus subtilis* subsp. *natto*	MH33−2	+	+	+	+	+
*Bacillus subtilis* subsp*. natto*	NBRC 3336	+	+	+	+	+
*Bacillus subtilis* subsp*. natto*	NBRC 16449	+	+	+	+	+
*Bacillus subtilis* subsp*. natto*	NBRC 3009	+	+	+	+	+
*Bacillus subtilis* subsp*. natto*	NBRC 3335	+	+	+	+	+
*Bacillus subtilis* subsp*. natto*	NBRC 3936	+	+	+	+	+
*Bacillus subtilis* subsp*. natto*	NBRC 13169	+	+	+	+	+
*Bacillus subtilis* subsp. *subtilis*	NBRC 3027	N.D.	++	+	N.D.	+
*Bacillus subtilis* subsp. *subtilis*	NBRC 3108	N.D.	N.D.	N.D.	N.D.	+
*Bacillus subtilis* subsp*. subtilis*	NBRC 3513	N.D.	N.D.	N.D.	N.D.	+
*Bacillus subtilis* subsp*. subtilis*	NBRC 12210	N.D.	++	N.D.	N.D.	+
*Bacillus subtilis* subsp*. subtilis*	NBRC 14140	N.D.	++	+	+	+
*Bacillus subtilis* subsp*. subtilis*	NBRC 110487	N.D.	++	N.D.	N.D.	+
*Bacillus subtilis* subsp. *subtilis*	NCIB 3610	N.D.	++	+	N.D.	+
*Bacillus subtilis* subsp. *subtilis*	Marburg 168	N.D.	++	N.D.	N.D.	+
*Bacillus subtilis* subsp. *spizizenii*	NBRC 101239	N.D.	++	N.D.	N.D.	+
*Bacillus subtilis* subsp. *spizizenii*	NBRC 101243	N.D.	N.D.	N.D.	N.D.	+
*Bacillus amyloliquefaciens* subsp. *amyloliquefaciens*	NBRC 3007	N.D.	N.D.	N.D.	N.D.	+
*Bacillus amyloliquefaciens* subsp*. amyloliquefaciens*	NBRC 101583	N.D.	N.D.	N.D.	N.D.	+
*Bacillus pumilus*	NBRC 3813	N.D.	N.D.	N.D.	N.D.	+
*Bacillus pumilus*	NBRC 12092	N.D.	N.D.	N.D.	N.D.	+
*Bacillus pumilus*	ATCC 14884	N.D.	N.D.	N.D.	N.D.	+
*Bacillus licheniformis*	NBRC 12200	N.D.	N.D.	N.D.	N.D.	+
*Bacillus licheniformis*	NBRC 109103	N.D.	N.D.	N.D.	N.D.	+
*Bacillus paranthracis*	ATCC 13061	N.D.	N.D.	N.D.	N.D.	+
*Bacillus paralicheniformis*	ATCC 12759	N.D.	N.D.	N.D.	N.D.	+

PCR results are indicated as follows: + , amplification detected; N.D., not detected (no amplification). For the *comQ* marker, the primer set comQ-F241/R831 was used for validation. For the *bioF* marker, + and ++ represent the detection of short and long amplicons, respectively.

### DNA extraction and quality control

Genomic DNA was extracted using the ISOPLANT kit (Nippon Gene Co., Ltd., Tokyo, Japan) according to the manufacturer’s instructions. DNA concentration and purity (*A*_260_/*A*_280_ ratio) were initially assessed using a BioSpec-nano spectrophotometer (Shimadzu Corp., Kyoto, Japan) for preliminary screening and conventional PCR assays. For qPCR applications, DNA concentrations were determined with higher precision using a Qubit 4 Fluorometer with the Qubit dsDNA HS Assay Kit (Thermo Fisher Scientific, Waltham, MA, USA). This dual-quantification strategy ensured both high purity—required for specificity assays—and stringent precision—necessary for the construction of standard curves and sensitivity analysis.

### Genome sequencing and assembly

Shotgun genome sequencing libraries were constructed for the nine *B. subtilis* strains newly sequenced in this study using the MGIEasy Fast FS Library Prep Set V2.0 (MGI Tech Co., Ltd., Shenzhen, China) and sequenced on a DNBSEQ-T7 platform as 150-bp paired-end reads. Raw reads were quality-filtered and adapter-trimmed using fastp (version 1.0.1) with default parameters, including a qualified quality Phred score threshold of 15 and an unfiltered base limit of 40% [23]. The quality-filtered reads were assembled *de novo* using SPAdes assembler (version 4.2.0) with the careful option enabled to minimize mismatches and short indels, utilizing a multi-k-mer strategy with k-mer lengths of 21, 33, 55, and 77 [24]. The draft genome sequences were annotated by DFAST [25]. The draft genome sequences were deposited in the EMBL/GenBank/DDBJ database under BioProject ID PRJDB398070. The detailed genomic metrics and accession numbers for each strain are listed in S1 Table.

### Genome-wide similarity assessment and phylogenetic analysis of the *comQXPA* locus

**Fig 1:**
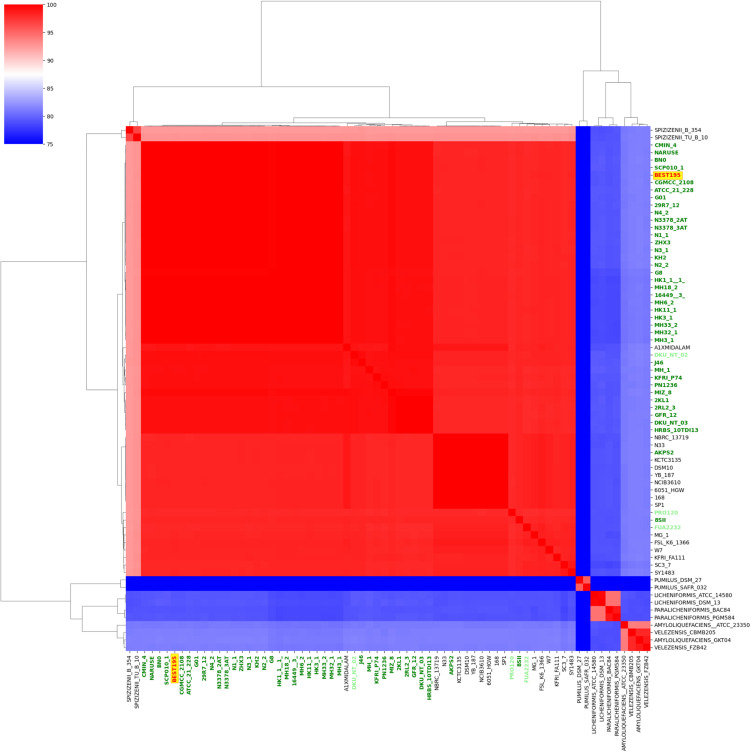
Genome-wide average nucleotide identity (ANI) heatmap and dendrogram analysis of the *Bacillus subtilis* group and closely related *Bacillus* species. The dendrograms on the top and left sides illustrate the hierarchical phylogenetic clustering based on genomic distances, driving the matrix organization. The color scale indicates pairwise ANI percentages ranging from 75% (blue) to 100% (red) across 70 bacterial genomes. Strains exhibiting 100% *comQ* sequence identity with the reference strain BEST195 are highlighted in dark green text (38 strains), whereas those exhibiting 98% or higher identity are indicated in light green text (3 strains). The reference strain BEST195 itself is indicated in red text with yellow shading.

To evaluate the genome-wide similarity across the species complex, average nucleotide identity (ANI) was calculated for all 70 bacterial genomes—encompassing the 9 *B. subtilis* strains newly sequenced in this study, 49 other *B. subtilis* group strains, and 12 closely related *Bacillus* species—using the FastANI tool within the Galaxy platform (https://usegalaxy.org/) with default parameters, employing the representative *subsp. natto* strain BEST195 as the reference genome. The resulting pairwise ANI matrix was subjected to hierarchical clustering analysis, and the genome-wide phylogenetic stratification was visualized as a clustered heatmap (Fig 1) using Python with the scipy.cluster.hierarchy and seaborn libraries.

Four distinct phylogenetic trees were constructed independently, each based on the amino acid sequence of an individual component of the *comQXPA* locus (ComQ, ComX, ComP, and ComA, respectively) from subsp. *natto* and its closely related species. In particular, these separate alignments were used to evaluate the phylogenetic relationships between subsp. *natto* BEST195 and other members of the *B. subtilis* species complex. Multiple sequence alignment was performed using MAFFT (version 7) with default parameters. Phylogenetic reconstruction was subsequently conducted in MEGA12 (version 12) [26] using the neighbor-joining (NJ) method based on the Poisson correction model. The reliability of the tree topology was evaluated via bootstrap analysis with 1,000 replicates. Accession numbers for all analyzed sequences are provided in S1 Table.

### Primer design

Specific primers were designed to target the *comQ* gene of *B. subtilis* subsp. *natto* BEST195. Two distinct primer sets were optimized: comQ-F241/R831 for general lineage validation via conventional PCR, and comQ-qF/qR for lineage-specific qPCR assays (S2 Table). To verify the primer binding sites and evaluate amplification specificity *in silico*, nucleotide sequence alignments were performed using MEGA12, encompassing the comprehensive dataset of 70 bacterial genomes assembled in this study. The primer design was optimized to ensure full complementarity across the target subsp. *natto* cohort while avoiding cross-reactivity with non-target reference sequences, such as *B. subtilis* subsp. *subtilis* 168 and *B. subtilis* subsp. *spizizenii* NBRC 101239.

### Conventional PCR amplification and specificity testing

The 16S rRNA gene, *comQ*, *bioF*, and IS elements (IS*4Bsu1* and IS*256Bsu1*) were amplified using PCR. Detailed information about the primers used for the PCR is provided in S2 Table. As described above, the primer set comQ-F241/R831 was utilized for the direct detection and validation of the *comQ* gene. To evaluate conventional markers, we used the *bioF-* and IS-specific primers described by Kimijima et al. [13]. The 16S rRNA gene served as an internal control, with universal primers 27F/1512R used for general specificity assays and 341F/907R for sensitivity assays. Reactions were prepared in a 20 µL total volume using EmeraldAmp MAX PCR Master Mix (Takara Bio Inc., Kusatsu, Japan). The thermal cycling profile consisted of 30 cycles of denaturation at 98 °C for 10 s, annealing at 60 °C for 30 s, and extension at 72 °C for 1 min. Each assay was performed in triplicate to ensure reproducibility. PCR products were analyzed via electrophoresis on a 1.5% (w/v) agarose gel and visualized after ethidium bromide staining under UV illumination. Gel images were captured and converted to grayscale using Adobe Photoshop 2020.

### qPCR and construction of a universal standard curve

qPCR was performed using the StepOne Real-Time PCR System (Thermo Fisher Scientific). Each 10 µL reaction mixture consisted of 5 µL of TB Green Premix Ex Taq II (Tli RNaseH Plus) (Takara Bio), 0.4 µM of each primer (comQ-qF/qR), and 1 µL of template DNA. To evaluate the selectivity of the assay against non-target lineages, 10 ng of genomic DNA from *B. subtilis* subsp. *subtilis* 168 or *B. subtilis* subsp. *spizizenii* NBRC 101239 was used per reaction. The fast-cycling protocol was as follows: initial denaturation at 95 °C for 30 s, followed by 40 cycles of denaturation at 95 °C for 5 s and annealing/extension at 60 °C for 30 s. A melting curve analysis (from 60 °C to 95 °C) was conducted to confirm amplicon specificity.

### DNA extraction from commercial *natto* matrices and viable cell enumeration

For real-time validation of the assay, three distinct brands of commercial *natto* (fermented soybean) products were purchased from a local supermarket. For each sample, 10 grains of *natto* were suspended in 5 mL of phosphate-buffered saline (PBS) to prepare the crude matrix extracts. Total genomic DNA was extracted from the resulting suspension using ISOPLANT as described previously. The isolated DNA samples were subjected to 10-fold serial dilutions (1^00^ to 10^−3^) and used as templates for qPCR evaluation. Concurrently, for enumerating viable cells, crude *natto* suspensions were serially diluted in PBS, plated onto BHI agar in triplicate, and incubated overnight at 37 °C. The resulting colonies were counted, and the viable cell counts (log_10_ CFU/mL) were expressed as the mean ± standard deviation (SD).

### Preparation of standard plasmid DNA

To construct the standard plasmid framework for absolute quantification, the *comQ* gene fragment was amplified from *B. subtilis* subsp. *natto* NBRC 16449 genomic DNA using the specific primer set comQ-F241/R831. The resulting PCR amplicon was cloned into the pMD20-T vector (Takara Bio) via TA cloning according to the manufacturer’s protocol. The concentration of the purified recombinant plasmid DNA was determined spectrophotometrically. The corresponding molecular copy number was subsequently calculated based on the total size of the plasmid construct (vector plus insert) using the following formula:


$$\begin{array}{c} Plasmidcopynumber(copies/tube)=\frac{\begin{array}{c} PlasmidDNAconcentration\left(\frac{g}{\mu L}\right)\times \text{6}.\text{0}\text{2}\text{2}\times {\text{1}\text{0}}^{\text{2}\text{3}}\times \text{1}\mu L/tube \end{array}}{Totalplasmidsize(bp)\times \text{6}\text{6}\text{0}g/mol/bp} \end{array}$$

This plasmid stock was used to generate a 10-fold serial dilution series, ranging from 2.86 × 10^3^ to 2.86 × 10^6^ copies per reaction, to construct the absolute quantification standard curve (*R*^2^ = 0.9973; efficiency = 94.2%). To determine the absolute *comQ* copy numbers in the *natto* extracts, the experimental quantification cycle (*Cq*) values obtained from the samples were plugged into the linear regression equation derived from this plasmid standard curve (*Cq* = slope × log_10_(copy number) + intercept), thereby converting the raw *Cq* metrics into absolute log copies per reaction tube.

### Data visualization and statistical analysis

Based on the phylogenetic relationships, established as described in section “Genome-wide similarity assessment and phylogenetic analysis of the *comQXPA* locus,” we statistically evaluated the evolutionary congruence within the *comQXPA* locus. Mantel tests were performed using the skbio.stats.distance library to calculate correlations between the amino acid distance matrices of the four proteins (ComQ, ComX, ComP, and ComA). The statistical significance of the correlation coefficient (*r*) was determined using a permutation test with 1,000 random shufflings.

To evaluate the evolutionary selective pressure acting on the quorum-sensing components, the rates of synonymous (*dS*) and nonsynonymous (*dN*) substitutions were calculated using MEGA12. This analysis encompassed 42 strains, consisting of the reference strain BEST195 and 41 strains exhibiting 98.0% or higher *comQ* sequence identity with BEST195. The *dN/dS* ratios were determined independently for each of the *comQ*, *comX*, and *comP* genes using the Nei–Gojobori method with the Jukes–Cantor correction. Furthermore, a codon-based *Z*-test for purifying selection (alternative hypothesis: *dN* < *dS*) was conducted in MEGA12 to establish the statistical significance of the selective constraints, and the corresponding *Z*-statistics and *p*-values were determined for each signaling component.

For qPCR validation, linear regression was conducted using scipy.stats [27] and scikit-learn [28] to determine the slope, y-intercept, and *R*^2^. Amplification efficiency (*E*) was calculated using the formula *E* = 10^(−1/slope)^ − 1.

The robustness of the assay against non-target DNA interference was evaluated via a two-way analysis of variance (two-way ANOVA) using the statsmodels library [29]. This model evaluated the statistical significance of the main effects (log-transformed DNA concentrations and background groups) as well as their interaction effect on the *Cq* values. Data visualization was performed using matplotlib [30] and seaborn [31]. *Cq* values are presented as mean ± SD from three independent replicates, except for the data of strain NBRC 16449 in S3 Fig, which are based on five independent replicates. A *p*-value <0.05 was considered statistically significant.

## Results

### High genomic similarity within the *B. subtilis* group

To establish a robust genomic foundation, we conducted a genome-wide ANI analysis encompassing 70 bacterial genomes: 9 *B. subtilis* strains newly sequenced in this study, 49 other *B. subtilis* group strains, and 12 closely related *Bacillus* species. Strains within the *B. subtilis* group, including both subsp. *natto* and subsp. *subtilis*, exhibited extremely high genomic similarity, with ANI values exceeding 98% relative to the representative *natto* strain BEST195 (Fig 1). This confirmed a high degree of genomic uniformity, consistent with their classification within the *B. subtilis* species complex.

### Phylogenetic stratification and co-evolutionary analysis of the *comQXPA* locus

In contrast to the overall genomic homogeneity, the *comQXPA* locus displayed profound sequence divergence. Bioinformatic analysis revealed a distinct stratification of the *B. subtilis* group based on the amino acid sequence identity of each signaling component (S1 Table). Beyond the reference strain BEST195, a primary cluster of 38 strains exhibited 100% identity for the QS activator ComQ, and three additional strains shared over 98.0% identity. Conversely, ComQ homology with subsp. *subtilis* strains (e.g., strain 168) dropped to approximately 35.5%. Similarly, the signaling peptide ComX and the sensor kinase ComP maintained high conservation within these 42 *natto*-lineage strains (100.0% and 99.0% or greater identity, respectively) but the homology dropped to 16.0% and 39.8% relative to strain 168. In sharp contrast, the response regulator ComA remained highly conserved across all subspecies (S1 Table).

**Fig 2:**
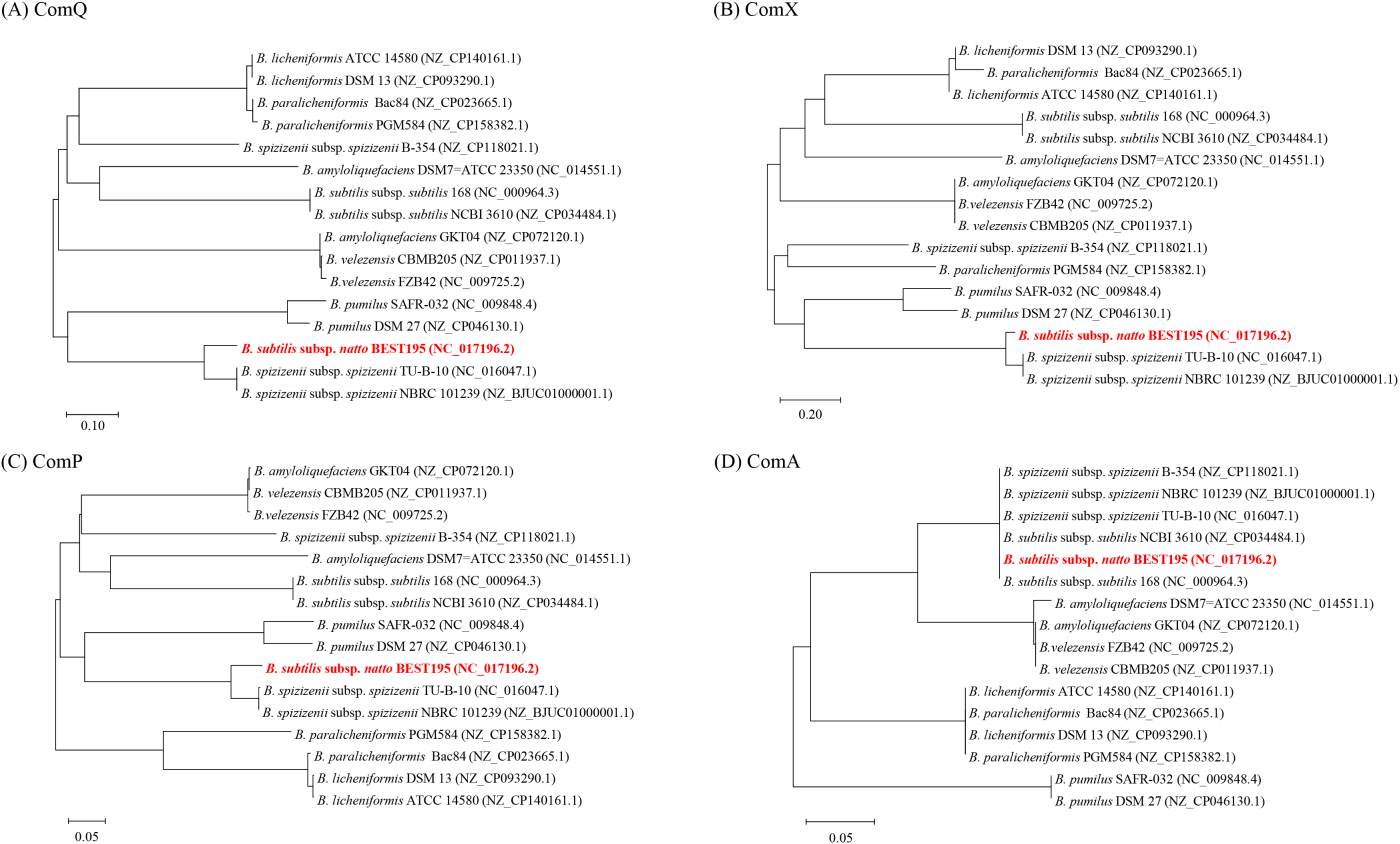
Phylogenetic analysis of the *comQXPA* quorum-sensing locus components. Neighbor-joining phylogenetic trees were constructed based on the amino acid sequences of (A) ComQ, (B) ComX, (C) ComP, and (D) ComA from various *Bacillus subtilis* groups. The evolutionary history was inferred using the Neighbor-Joining method based on the Poisson correction model, and the reliability of the tree topology was evaluated via bootstrap analysis with 1,000 replicates. The subsp. *natto* BEST195 is highlighted in red to represent the target functional lineage. Numbers in parentheses indicate NCBI accession numbers. Scale bars represent the number of amino acid substitutions per site.

This stratification was visually confirmed using neighbor-joining phylogenetic trees, where ComQ, ComX, and ComP from strain BEST195 formed a distinct monophyletic cluster that was well-separated from other subspecies (Fig 2A-C), whereas ComA showed no lineage-specific clustering (Fig 2D).

**Fig 3:**
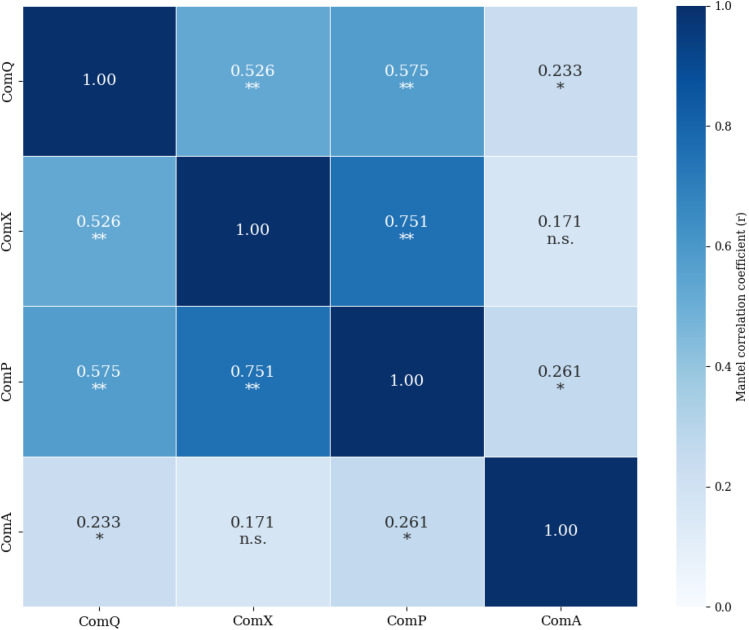
Correlation heatmap of the Mantel test among the *comQXPA* quorum-sensing locus components. The heatmap displays Mantel correlation coefficients (*r*) derived from the amino acid sequence distance matrices of ComQ, ComX, ComP, and ComA. The color gradient (blues) represents the strength of the correlation, with *r* values ranging from 0.0 to 1.0. Statistical significance was determined based on *p*-values calculated using the permutation tests (**, *p* < 0.01; *, *p* < 0.05; and n.s., not significant).

To evaluate whether these components function as an integrated system, we assessed their evolutionary coupling. Mantel tests confirmed strong and statistically significant correlations among ComQ, ComX, and ComP (Mantel *r* = 0.526–0.751, *p* < 0.01; Fig 3), indicating tight co-evolution as a functional unit to maintain signaling specificity. In contrast, ComA showed no evolutionary correlation with the other components (Fig 3), confirming its role as a universal, independent regulator.

### Co-occurrence of *comQ* with known *natto*-specific genetic markers

We next evaluated the diagnostic distribution of *comQ* against established genetic signatures of the *natto* lineage (S1 Table). Among the 38 core strains (100% *comQ* identity), we observed a high rate of co-occurrence, with the majority harboring BEST195-type sequences for *bioF*, *bioW*, *fliF*, and IS elements. However, this co-occurrence was incomplete in 15 atypical isolates (e.g., Miz-8 and PN1236), which exhibited discrepancies or deletions in one or more conventional markers (S1 Table). Remarkably, even in these variants where traditional markers were absent or diverged, both *comQ* and *comX* remained 100% conserved, indicating that *comQ* provides an evolutionary robustness that eliminates the risk of false-negative identification.

To investigate the evolutionary drivers underlying this conservation, we calculated the *dN/dS* ratio across the 42 natto-lineage strains (Table 2). The *comQ* gene exhibited a significantly low *dN/dS* ratio (0.10) and a significant negative *Z*-statistic (−1.68, *p* < 0.05). This indicates that *comQ* is under strong purifying selection. While *comX* remained sequence-invariant and *comP* reflected neutral evolution (*dN/dS* = 0.36), the intense evolutionary constraint on *comQ* suppresses non-synonymous mutations to preserve quorum-sensing integrity. Consequently, *comQ* serves as an exceptionally stable genomic anchor, which renders it the highly reliable molecular target for diagnostic assay development.

**Table 2:** Evolutionary constraints on the *comQXP* quorum-sensing locus in subsp. *natto.*

Gene	Identity (%)	*dN/dS*	*Z*-stat	Interpretation
*comQ*	98.5–100	0.1	−1.68	Strong purifying selection
*comX*	100	Invariant	0	Highly conserved
*comP*	99.2–100	0.36	0.92	Neutral evolution

### Design and *in silico* PCR validation of *comQ*-targeted primers

We designed two primer sets targeting the conserved regions of *comQ* in strain BEST195: comQ-F241/R831 for conventional PCR and comQ-qF/qR for qPCR. Alignment analysis revealed multiple nucleotide substitutions in non-*natto* subspecies (subsp. *subtilis* 168 and subsp. *spizizenii* NBRC 101239), particularly at the 3′ terminus, which is critical for polymerase extension (S1 Fig). *In silico* PCR simulations achieved 100% accuracy across the 70 genomes (S3 Table); all 42 *natto*-lineage strains were predicted to yield positive amplicons due to identical primer-binding sites, whereas all non-*natto* strains were predicted to be negative *in silico*.

### Specificity, inclusivity, and sensitivity of the *comQ*-targeted PCR assay

**Fig 4:**
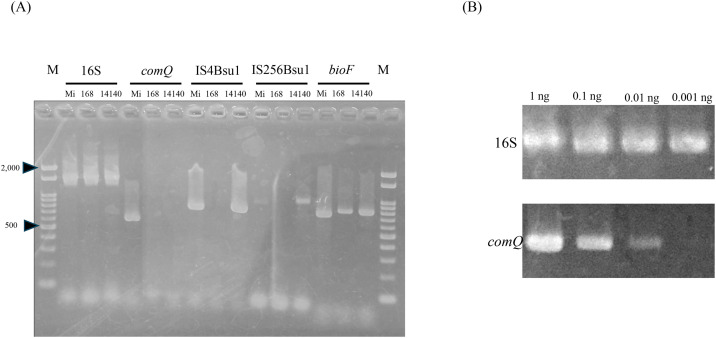
Specificity and sensitivity of the PCR assays using multiple primer sets. (A) Comparison of specificity between the *comQ* and conventional genetic markers. PCR amplification was performed targeting the 16S rRNA gene, *comQ*, IS*4Bsu1*, IS*256Bsu1*, and *bioF*. The tested strains included subsp. *natto* Miyagino (Mi), *Bacillus subtilis* subsp. *subtilis* 168, and *B. subtilis* subsp. *subtilis* NBRC 14140. Lane M: Gene-Ladder 100 (Nippon Gene Co., Ltd., Tokyo, Japan); black triangles indicate 2,000 and 500 bp. (B) Detection sensitivity of the *comQ* PCR assay. A 10-fold serial dilution of genomic DNA from subsp. *natto* Miyagino (1–0.001 ng per reaction) was used as a template. The 16S rRNA gene was used as a control. Gel images were converted to grayscale and adjusted for contrast using Adobe Photoshop 2020. All lanes in (B) originated from the same gel. The images shown are representative results from three independent experiments.

Laboratory experiments using 32 bacterial strains empirically validated the bioinformatic predictions (Table 1, Fig 4A). The comQ-F241/R831 primer set demonstrated 100% inclusivity, yielding positive results for all 18 *subsp. natto* strains (Table 1), with specific amplification visually confirmed via agarose gel electrophoresis for 13 representative strains (S2 Fig). In contrast, conventional *bioF* and IS-element markers caused cross-reactivity or non-specific bands in several non-target strains, including subsp. *subtilis* 168 and NBRC 14140 (Fig 4A). The *comQ* assay showed complete exclusivity (no detection) across all 19 non-target species, providing a clear presence/absence result. The conventional PCR assay also showed a reliable limit of detection (LOD) of 0.01 ng of template DNA per reaction (approximately 2.2 × 10^3^ genomic copies; Fig 4B).

### Quantitative performance, specificity, and robustness of the qPCR assay

The *comQ*-qF/qR qPCR assay demonstrated high specificity and quantitative performance. When evaluated against the closest phylogenetic neighbors, the target strain NBRC 16449 yielded a mean *Cq* of 19.53 (S3A Fig), whereas non-target strains 168 and NBRC 101239 exhibited delayed *Cq* values of 33.18 and 32.41, respectively (S4 Table). This *ΔCq* of approximately 13 cycles represents a > 8,000-fold discrimination efficiency against non-target orthologs. Melting curve analysis confirmed a single, sharp target dissociation peak (*Tm* = 74.95 °C), distinctly separated from non-target profiles (S3 Fig, S4 Table).

To establish the universal applicability and analytical sensitivity of the *comQ*-targeted qPCR assay, we constructed standard curves using genomic DNA from three representative strains, Miyagino, Naruse, and NBRC 16449. The assay employed a 10-fold serial dilution series ranging from 1.0 ng to 0.0001 ng of template DNA per reaction.

**Fig 5:**
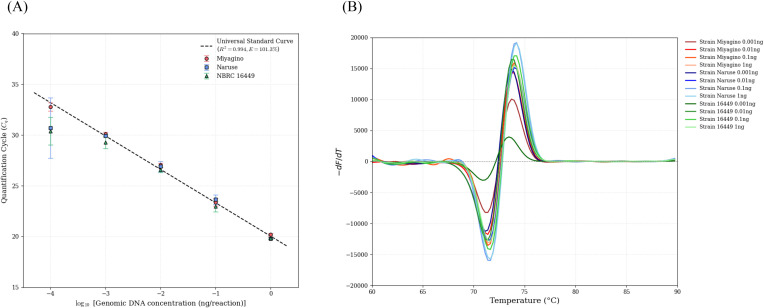
Standard curve and melting curve analysis of the *comQ* qPCR assay. (A) Universal standard curve generated from three *Bacillus subtilis* subsp. *natto* strains. The quantification cycle (*Cq*) values are plotted against the log–log-transformed genomic DNA concentration (ranging from 1.0 ng to 0.0001 ng per reaction) for subsp. *natto* Miyagino, Naruse, and NBRC 16449. The dashed line represents the linear regression calculated from the combined data from all three strains. (B) Melting curve profiles for the sensitivity assay. The derivative melting curve plot (*-dF/dT* vs. temperature) shows the *Tm* profiles of the amplicons generated from the three *natto* strains at varying template concentrations. All data represent the mean values from three independent replicates. Error bars in (A) indicate the standard deviation (SD).

The assay exhibited excellent linearity and high reproducibility across four orders of magnitude (1.0–0.001 ng) for all three strains (Fig 5). The composite standard curve showed a high coefficient of determination (*R*^2^ = 0.994) and an optimal amplification efficiency (*E*) of 101.3%. Within this linear range, *Cq* values were nearly identical across the three strains at each concentration (S4 Table). This minimal interstrain variance confirms that the *comQ* marker provides consistent quantitative results regardless of the specific subsp*. natto* strain analyzed.

**Fig 6:**
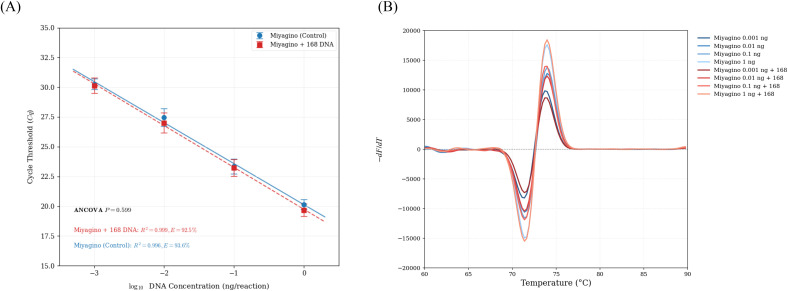
Robustness of the *comQ*-targeted qPCR assay against background genomic DNA. (A) Standard curves for *Bacillus subtilis* subsp. *natto* Miyagino genomic DNA (0.001–1.0 ng per reaction) in the absence (blue circles) and presence (red square) of 10 ng of genomic DNA from the nontarget strain *B. subtilis* subsp. *subtilis* 168. Lines represent the linear regression models for each condition. (B) Melting curve analysis of the qPCR products obtained from the samples in (A). For (A), each data point represents the mean ± standard deviation for triplicate reactions (*n* = 3).

To test matrix robustness, genomic DNA from strain Miyagino (1.0–0.001 ng) was quantified in the presence of a 10-fold excess (10 ng) of non-target strain 168 background DNA (Fig 6). At the lowest detection limit (0.001 ng), this background represents a 10,000-fold excess of non-target genetic material. Nonspecific background amplification remained negligible (*Cq* = 32.76 ± 1.32). For instance, 1.0 ng of target DNA yielded a *Cq* of 20.15 ± 0.40 with dH_2_O and 19.68 ± 0.54 with the 168 background DNA. Two-way ANOVA confirmed no significant background-induced differences in *Cq* values (Table 3). The regression slope was stable at −3.44, corresponding to an amplification efficiency (*E*) of 95.3%, with a consistent *Tm* peak (74.8–74.9 °C).

**Table 3:** Two-way ANOVA for the effects of subsp. *natto* Miyagino DNA concentration and *Bacillus subtilis* 168 DNA background on quantification cycle (*Cq*) values.

Source of Variation	df	Sum of Squares	Mean Square	*F*-value	*p*-value
Log DNA concentration	3	364.161	121.387	299.36	<0.001
Background Groups	1	0.479	0.479	1.18	0.293
Interaction (Conc × Group)	3	0.18	0.06	0.15	0.93
Residuals (Error)	16	6.488	0.405	–	–

Data are presented based on three independent experiments.

### Absolute quantification of *comQ* in commercial *natto* extracts

A standard curve for recombinant plasmid carrying the *comQ* fragment demonstrated high linearity (*R*^2^ = 0.9973) and efficiency (*E* = 94.2%, S5 Fig) of the assay. This assay was applied to total DNA extracted from three commercial *natto* brands (Table 4) with viable cell counts ranging from 8.73 to 8.82 log_10_ CFU/mL. Across a 10-fold serial dilution series (1^00^ to 10^−3^), mean *Cq* values increased in a predictable, dilution-dependent manner. Stable mean *Tm* values (74.51–75.10 °C) confirmed the absence of matrix-derived inhibition. At 1^00^ dilution, the calculated copy number for *comQ* ranged from 6.46 to 6.63 log_10_ copies/tube and decreased systematically with each dilution step. These metrics validate the utility of this plasmid-standardized qPCR assay for precise, culture-independent monitoring of the *natto* lineage within complex commercial matrices.

**Table 4:** Quantitative performance and matrix tolerance of the *comQ*-targeted qPCR assay in commercial natto extracts.

Sample	Viable cell count (log_10_ CFU/mL)	Dilution	Mean *Cq* ± SD	Mean *Tm* ± SD	Calculated *comQ* copy number (log_10_ copies/tube)
Natto extract 1	8.73 ± 0.08	10^0^	16.02 ± 0.24	75.10 ± 0.09	6.63
		10 ^− 1^	19.14 ± 0.36	75.10 ± 0.09	5.75
		10 ^− 2^	23.43 ± 0.75	74.71 ± 0.15	4.54
		10 ^− 3^	26.21 ± 0.36	74.95 ± 0.09	3.75
Natto extract 2	8.74 ± 0.11	10^0^	16.02 ± 0.04	74.95 ± 0.23	6.63
		10 ^− 1^	19.42 ± 0.90	74.95 ± 0.23	5.67
		10 ^− 2^	23.59 ± 0.53	74.95 ± 0.17	4.49
		10 ^− 3^	26.86 ± 0.58	74.55 ± 0.15	3.57
Natto extract 3	8.82 ± 0.16	10^0^	16.61 ± 0.05	74.95 ± 0.23	6.46
		10 ^− 1^	19.43 ± 0.61	74.80 ± 0.09	5.67
		10 ^− 2^	23.22 ± 0.76	74.75 ± 0.09	4.59
		10 ^− 3^	26.44 ± 1.29	74.51 ± 0.30	3.68

## Discussion

In this study, we developed and validated a qPCR assay targeting the *comQ* gene for the rapid, culture-independent, and specific identification of *B. subtilis* subsp. natto. Although tracking this lineage is essential to the food biotechnology industry, universal markers often lack sufficient resolution. This limitation stems from the high genomic similarity within the *B. subtilis* species complex [1,9,10], which frequently compromises standard housekeeping or phylogenetic loci [11,12]. By leveraging an expanded comparative dataset of 70 genomes and absolute quantification metrics, we demonstrate the robustness of *comQ* as a genomic anchor in resolving subspecies-level stratification despite genome-wide homogeneity.

Our analysis revealed a remarkable conservation of the *comQXPA* locus across diverse ecological origins. Notably, eight analyzed subsp. *natto* genomes originated from honey [13], yet their *comQXPA* sequences exhibited 100% identity with those isolated from fermented soybeans. This high stability suggests that the *comQ*-mediated QS system is a core, nonredundant component of the *natto* genetic identity, which has remained evolutionarily static across disparate isolation sources, ensuring reliable detection without false negatives.

While genome-wide ANI analysis confirmed high uniformity (>98%) across the *B. subtilis* complex, the *comQXPA* operon stands out as a hotspot of diversification [20,21], where *comQ* determines “pherotype” specificity [20,32]. Multi-locus *dN/dS* analysis yielded a remarkably low ratio (0.10) and a significant negative *Z*-statistic (−1.68, Table 2), providing empirical evidence of intense purifying selection. This evolutionary constraint is likely driven by the ecological requirement to maintain a functional pherotype system that coordinates cell density-dependent traits—such as γ-PGA synthesis, nattokinase production, extracellular enzyme secretion, and biofilm formation—that collectively define the *natto* phenotype [1,32,33]. Furthermore, Mantel tests confirmed strong correlations among ComQ, ComX, and ComP (Mantel *r* = 0.526–0.751, *p* < 0.01; Fig 3), indicating tight co-evolution to preserve signaling fidelity. Thus, *comQ* copy number serves as a functional proxy for the fermentation potential of the population within a given matrix [18].

To establish a genetic framework for quantifying subsp. *natto*, traditional loci, such as the *bio* operon (*bioF* and *bioW*) and *fliF*, are common candidate targets [13–15]. However, genomic screening of 42 strains with >98.0% *comQ* identity revealed the failure of 15 strains to meet the 99.0% identity threshold for at least one traditional locus. Cross-referencing these with public pangenomic resources showed that 10 belonged to the primary “*natto*-related group,” whereas strain PRO120 was positioned outside this cluster based on macro-genomic distance metrics [16]. This demonstrates that the *comQ* assay provides a complementary functional perspective, effectively capturing such strains that, despite their macro-genomic divergence, retain the essential QS architecture for fermentation.

In contrast to the robustness of the *comQ* locus, relying on *bio* or *fliF* introduces technical risks of false negatives or cell-equivalent underestimation due to localized truncations [16]. Similarly, alternative IS-based markers, such as IS*4Bsu1* and IS*256Bsu1*, lack subspecies exclusivity and vary inconsistently among strains [15]. Notably, these mobile elements can induce genetic instability in key industrial traits [34,35], further highlighting the limitations of conventional region-specific targeting.

Despite the phylogenetic proximity and shared pherotype trends between subsp. *natto* and *Bacillus spizizenii* [20,21], our platform clearly differentiates the *natto* clade from *B. spizizenii* NBRC 101239, its closest taxonomic neighbor [36,37]. By targeting sublineage-specific variations, our assay achieved a significant *ΔCq* of approximately 13 cycles against strain NBRC 101239 (S3A Fig). Traditional frameworks require precise fragment size discrimination (*bioF*) [14,15] or rely on volatile elements (IS) [1,15]. In contrast, *comQ* offers a highly specific detection window rooted in profound sequence divergence [1, 38], ensuring comprehensive coverage and high quantification fidelity even from its closest taxonomic relatives.

Compared with conventional PCR, the qPCR assay developed in this study demonstrated enhanced analytical capability. While the conventional method exhibited a gel-based LOD of 0.01 ng (Fig 4B), the qPCR platform established a strict limit of quantification (LOQ) of 0.001 ng of genomic DNA based on statistical reproducibility (Fig 5A and S4 Table). Although these metrics are derived from different criteria, the fact that the qPCR LOQ is already 10-fold lower than the conventional LOD logically underscores the vastly superior analytical sensitivity and dynamic range of the qPCR framework. To bridge this sensitivity with practical applications, a standard curve for recombinant plasmid carrying the *comQ* fragment was introduced (*R*^2^ = 0.9973, y = −3.468x + 38.811, S5 Fig), utilizing robust statistical and visualization environments. When applied to three commercial *natto* brands (Table 4), the calculated *comQ* copies systematically decreased from 6.46 to 6.63 log_10_ copies/tube at the undiluted level across a 10-fold dilution series (1^00^ to 10^−3^), demonstrating robust quantification at trace levels.

The assay also addressed genomic interference under rigorous conditions involving up to a 10,000-fold excess of non-target DNA. Two-way ANOVA confirmed that background DNA from strain 168 did not significantly shift the *Cq* values (*p* = 0.293, Table 3), demonstrating over 8,000-fold selectivity. This resistance to competitive inhibition justifies the use of SYBR Green I over probe-based methods because selectivity is inherently established during primer extension. Furthermore, a stable *Tm* profile (approximately 74.5–75.1 °C) serves as an indispensable secondary validation of product purity, offering a level of assay transparency unavailable for the binary fluorescent signals from probes, as highlighted by the MIQE guidelines [39] and established melting curve principles [40].

Beyond industrial quality control, this platform is applicable to clinical microbiology. Although generally recognized as safe, subsp. *natto* has recently been implicated in opportunistic bacteremia and sepsis [8]. Our assay enables direct, culture-independent verification of subsp. *natto* in complex clinical matrices, supporting both fermentation integrity and public health surveillance.

From a practical perspective, our findings confirm that *comQ* serves as a stable, single-copy marker across the vast majority of the *natto* group [16], with its high sequence identity and consistent standard curves accurately reflecting actual cell equivalents without the copy-number variations often associated with alternative markers [15,34]. Our genomic screening identified only a single structural exception in strain AKPS2, which harbors two identical copies of *comQ* (S3 Table, S4 Fig). Because both copies exhibit 100% primer-binding identity, amplification is highly efficient, preventing false negatives. Although such variants could slightly overestimate cell equivalents in rare instances, this structural regularity generally offers a rapid, cost-effective alternative to conventional, labor-intensive MLST [9] or whole-genome sequencing approaches [15,16].

Because DNA detection alone does not necessarily prove the intactness of the full quorum-sensing pathway or actual fermentation performance, further phenotypic validation is necessary to clarify the correlation between genotypic abundance and industrial capacity in solid-state matrices. Furthermore, a previous study reported that IS*4Bsu1*-mediated insertion into *comP* can occur in *B. subtilis* subsp. *natto*, leading to genetic instability of γ-PGA production [41]. This indicates that while the *comQXPA* system is highly conserved within the sampled strains, it is not completely resistant to insertional disruption. Therefore, *comQ* should be regarded as a highly useful diagnostic and quantitative proxy within the analyzed dataset, rather than a definitive lineage-defining taxonomic marker. Finally, although our optimized standard curve achieved a low LOQ, tracking trace amounts in specific environmental or complex processing settings may face limitations. For such applications, integrating pre-enrichment protocols, probe-based TaqMan chemistries, or digital PCR platforms could further enhance the analytical sensitivity and robustness of the assay [39, 40].

## Supporting information

S1 Table Comprehensive genomic comparison, *comQXPA* locus conservation, and molecular marker profiles among *Bacillus subtilis* group and related species. Detailed bioinformatic profiles of the 70 analyzed bacterial genomes, including *Bacillus subtilis* group strains and closely related *Bacillus* species. Average nucleotide identity (ANI) values and aligned fragment counts were calculated relative to the representative *B. subtilis* subsp. *natto* strain BEST195. Amino acid sequence identities (%) and sequence lengths (aa) are provided for each component of the quorum-sensing locus (ComQ, ComX, ComP, and ComA). The groupings indicated in the column “Analyzed by Seki & Nagano (2025)” reflect the taxonomic and functional classifications (such as Narrow-sense natto group, natto-related group, and Not natto group) adapted from the comparative genomic framework established by Seki and Nagano [16]. The rightmost columns indicate the presence (+) or absence (−) of conventional natto-specific genetic markers (*bioF*, *bioW*, *fliF*, IS*4Bsu1*, and IS*256Bsu1*), defined by a sequence identity threshold of ≥99% relative to strain BEST195. Gray shading visually highlights the presence (+) of these target markers to demonstrate their co-occurrence and discrepancy patterns across the lineages.
(XLSX)

S2 Table List of primers used in this study. (XLSX)

S3 Table *In silico* copy number analysis and PCR validation of the *comQ* gene across various *Bacillus* strains. This table presents the predicted copy numbers of the *comQ* gene and the *in silico* PCR electronic amplification profiles using two diagnostic primer sets (comQ-F241/R831 and comQ-qF/qR). The copy number of *comQ* represents the number of genetic loci exhibiting ≥98% sequence identity relative to the representative *Bacillus subtilis* subsp. *natto* strain BEST195. For the *in silico* PCR analysis, a plus sign (+) denotes that both the forward and reverse primers share a 100% perfect match with their respective target binding sites, predicting positive electronic amplification. A zero (0) indicates a lack of predicted amplification due to nucleotide mismatches or the absence of the target locus.
(XLSX)

S4 Table Quantitative performance and melting temperature analysis of the *comQ*-targeted qPCR assay for *Bacillus subtilis* subsp. *natto* and related strains. (XLSX)

S1 Fig Alignment of the *comQ* gene sequences from representative *Bacillus subtilis* strains for primer designing. Multiple sequence alignment of the *comQ* gene from *B. subtilis* subsp. *natto* BEST195, *B. subtilis* subsp. *subtilis* 168, and *B. subtilis* subsp. *subtilis* NBRC 101239. Red letters indicate mismatched nucleotides relative to the BEST195 sequence. Arrows indicate the primer binding sites and directions.
(TIF)

S2 Fig PCR amplification of the *comQ* gene from various *Bacillus subtilis* (*natto*) strains. PCR products were amplified using the primer set comQ-241F and comQ-831R and analyzed via agarose gel electrophoresis. Lanes 1–13, NBRC 3013, Miyagino, Takahashi, Naruse, HK1−1, HK11−1, HK3−1, NBRC 3336, NBRC 16449, NBRC 3009, NBRC 3335, NBRC 3936, and NBRC 13169. Lane M, Gene-Ladder 100 (Nippon Gene Co., Ltd., Tokyo, Japan); black triangles indicate 2,000 and 500 bp. Gel images were converted to grayscale and adjusted for contrast using Adobe Photoshop 2020. The images shown are representative results from three independent experiments.
(TIF)

S3 Fig Specificity of the qPCR assay for the *Bacillus subtilis* subsp. *natto*-specific target. (A) Difference in *Cq* values (*ΔCq*) between the target and nontarget strains. (B) Derivative melting curve analysis (−*dF/dT*) showing the distinct melting peaks for the target and nontarget strains. The green, blue, and red lines represent the subsp. *natto*-specific target (NBRC 16449), *B. subtilis* 168, and *B. subtilis* NBRC 101239, respectively. Both (A) and (B) were generated from the mean values from three independent experiments, with the exception of the data for strain NBRC 16449, which were based on five independent experiments.
(TIF)

S4 Fig Genomic organization of the *comQXPA* quorum-sensing locus and its structural duplication in *Bacillus subtilis* subsp. *natto.* Comparative schematic representation of the *com* gene clusters among the reference strain BEST195 and the two duplicated loci in strain AKPS2 (designated as AKPS2−1 and AKPS2−2). Arrows indicate the position, length, and transcriptional direction of each gene. Red arrows represent the core quorum-sensing components (*comA*, *comP*, *comX*, *comQ*) and *degQ*. Blue and gray arrows indicate the *tlpA* pseudogene and the uncharacterized gene, respectively. Genomic coordinates (kb) are indicated above the horizontal line for each locus.
(TIF)

S5 Fig Standard curve of the *comQ*-targeted qPCR assay generated using a recombinant plasmid standard. The quantification cycle (*Cq*) values are plotted against the log_10_-transformed copy numbers of the *comQ* gene fragment per reaction tube. The dotted red line represents the linear regression model derived from the experimental data. Each data point represents the mean value obtained from three independent replicates (*n* = 3), with error bars indicating the standard deviation (SD).
(TIF)

## References

[1] Nishito Y, Osana Y, Hachiya T, Popendorf K, Toyoda A, Fujiyama A, et al. Whole genome assembly of a natto production strain Bacillus subtilis natto from very short read data. BMC Genomics. 2010;11:243. doi: 10.1186/1471-2164-11-243 20398357 PMC2867830

[2] Inatsu Y, Nakamura N, Yuriko Y, Fushimi T, Watanasiritum L, Kawamoto S. Characterization of Bacillus subtilis strains in Thua nao, a traditional fermented soybean food in northern Thailand. Lett Appl Microbiol. 2006;43(3):237–42. doi: 10.1111/j.1472-765X.2006.01966.x 16910925

[3] Elbanna K, Alsulami FS, Neyaz LA, Abulreesh HH. Poly (γ) glutamic acid: a unique microbial biopolymer with diverse commercial applicability. Front Microbiol. 2024;15:1348411. doi: 10.3389/fmicb.2024.1348411 38414762 PMC10897055

[4] Afzaal M, Saeed F, Islam F, Ateeq H, Asghar A, Shah YA, et al. Nutritional Health Perspective of Natto: A Critical Review. Biochem Res Int. 2022;2022:5863887. doi: 10.1155/2022/5863887 36312453 PMC9616652

[5] Zhang J, Bilal M, Liu S, Zhang J, Lu H, Luo H, et al. Isolation, Identification and Antimicrobial Evaluation of Bactericides Secreting Bacillus subtilis Natto as a Biocontrol Agent. Processes. 2020;8(3):259. doi: 10.3390/pr8030259

[6] Yokoyama T, Nakamura T, Kimijima M, Mandokoro K, Tokumaru M, Takatsuka A, et al. Subtilisin NAT, a subtilisin-like serine protease present in fermented soybean “natto” extract, inhibits <i>Streptococcus mutans</i> biofilm formation. FSTR. 2021;27(3):537–42. doi: 10.3136/fstr.27.537

[7] Fujii K, Kubo Y, Noguchi T, Tobita K. Effects of Bacillus subtilis Natto Strains on Antiviral Responses in Resiquimod-Stimulated Human M1-Phenotype Macrophages. Foods. 2023;12(2):313. doi: 10.3390/foods12020313 36673407 PMC9858497

[8] Sada RM, Yamamoto G, Hamaguchi S, Kuroda E, Okura A, Cai M, et al. The majority of *Bacillus subtilis* strains isolated from blood cultures were derived from traditional Japanese fermented soybeans natto: A single-center retrospective study. Open Forum Infect Dis. 2025;12:ofaf574. doi: 10.1093/ofid/ofaf574 PMC12464813 41018695

[9] Kubo Y, Rooney AP, Tsukakoshi Y, Nakagawa R, Hasegawa H, Kimura K. Phylogenetic analysis of Bacillus subtilis strains applicable to natto (fermented soybean) production. Appl Environ Microbiol. 2011;77(18):6463–9. doi: 10.1128/AEM.00448-11 21764950 PMC3187134

[10] Kamada M, Hase S, Sato K, Toyoda A, Fujiyama A, Sakakibara Y. Whole genome complete resequencing of Bacillus subtilis natto by combining long reads with high-quality short reads. PLoS One. 2014;9(10):e109999. doi: 10.1371/journal.pone.0109999 25329997 PMC4199626

[11] Heo J, Kim J-S, Hong S-B, Park B-Y, Kim S-J, Kwon S-W. Genetic marker gene, recQ, differentiating Bacillus subtilis and the closely related Bacillus species. FEMS Microbiol Lett. 2019;366(16):fnz172. doi: 10.1093/femsle/fnz172 31675066

[12] Liu Y, Štefanič P, Miao Y, Xue Y, Xun W, Zhang N, et al. Housekeeping gene gyrA, a potential molecular marker for Bacillus ecology study. AMB Express. 2022;12(1):133. doi: 10.1186/s13568-022-01477-9 36287351 PMC9606167

[13] Kimijima M, Mandokoro K, Ichikawa Y, Tokumaru M, Narisawa N, Takenaga F. Isolation and characterization of <i>Bacillus subtilis</i> from commercially available honey and its application in natto fermentation. FSTR. 2022;28(3):267–73. doi: 10.3136/fstr.fstr-d-21-00312

[14] Sasaki M, Kawamura F, Kurusu Y. Genetic analysis of an incomplete bio operon in a biotin auxotrophic strain of Bacillus subtilis natto OK2. Biosci Biotechnol Biochem. 2004;68(3):739–42. doi: 10.1271/bbb.68.739 15056910

[15] Kamada M, Hase S, Fujii K, Miyake M, Sato K, Kimura K, et al. Whole-Genome Sequencing and Comparative Genome Analysis of Bacillus subtilis Strains Isolated from Non-Salted Fermented Soybean Foods. PLoS One. 2015;10(10):e0141369. doi: 10.1371/journal.pone.0141369 26505996 PMC4624242

[16] Seki K, Nagano Y. Conserved accessory genes link a phylogenetically distinct Bacillus subtilis strain from Indian bekang to the Japanese natto clade. Sci Rep. 2025;15(1):43097. doi: 10.1038/s41598-025-29683-y 41407778 PMC12712070

[17] Xu X, Kovács ÁT. How to identify and quantify the members of the *Bacillus* genus? Environ Microbiol. 2024;26: e16593. doi: 1111/1462-2920.16593 10.1111/1462-2920.16593 38383138

[18] Na H-E, Heo S, Kim T, Lee G, Lee J-H, Jeong D-W. ComQXPA quorum-sensing systems contribute to enhancing the protease activity of Bacillus velezensis DMB05 from fermented soybeans. Int J Food Microbiol. 2023;401:110294. doi: 10.1016/j.ijfoodmicro.2023.110294 37336024

[19] Hoffmann K, Wollherr A, Larsen M, Rachinger M, Liesegang H, Ehrenreich A, et al. Facilitation of direct conditional knockout of essential genes in Bacillus licheniformis DSM13 by comparative genetic analysis and manipulation of genetic competence. Appl Environ Microbiol. 2010;76(15):5046–57. doi: 10.1128/AEM.00660-10 20543043 PMC2916460

[20] Stefanic P, Mandic-Mulec I. Social interactions and distribution of Bacillus subtilis pherotypes at microscale. J Bacteriol. 2009;191(6):1756–64. doi: 10.1128/JB.01290-08 19114482 PMC2648371

[21] Tortosa P, Logsdon L, Kraigher B, Itoh Y, Mandic-Mulec I, Dubnau D. Specificity and genetic polymorphism of the Bacillus competence quorum-sensing system. J Bacteriol. 2001;183(2):451–60. doi: 10.1128/JB.183.2.451-460.2001 11133937 PMC94899

[22] Dogsa I, Choudhary KS, Marsetic Z, Hudaiberdiev S, Vera R, Pongor S, et al. ComQXPA quorum sensing systems may not be unique to Bacillus subtilis: a census in prokaryotic genomes. PLoS One. 2014;9(5):e96122. doi: 10.1371/journal.pone.0096122 24788106 PMC4008528

[23] Chen S, Zhou Y, Chen Y, Gu J. fastp: an ultra-fast all-in-one FASTQ preprocessor. Bioinformatics. 2018;34(17):i884–90. doi: 10.1093/bioinformatics/bty560 30423086 PMC6129281

[24] Prjibelski A, Antipov D, Meleshko D, Lapidus A, Korobeynikov A. Using SPAdes De Novo Assembler. Curr Protoc Bioinformatics. 2020;70(1):e102. doi: 10.1002/cpbi.102 32559359

[25] Tanizawa Y, Fujisawa T, Nakamura Y. DFAST: a flexible prokaryotic genome annotation pipeline for faster genome publication. Bioinformatics. 2018;34(6):1037–9. doi: 10.1093/bioinformatics/btx713 29106469 PMC5860143

[26] Kumar S, Stecher G, Suleski M, Sanderford M, Sharma S, Tamura K. MEGA12: Molecular Evolutionary Genetic Analysis Version 12 for Adaptive and Green Computing. Mol Biol Evol. 2024;41(12):msae263. doi: 10.1093/molbev/msae263 39708372 PMC11683415

[27] Virtanen P, Gommers R, Oliphant TE, Haberland M, Reddy T, Cournapeau D, et al. SciPy 1.0: fundamental algorithms for scientific computing in Python. Nat Methods. 2020;17(3):261–72. doi: 10.1038/s41592-019-0686-2 32015543 PMC7056644

[28] Pedregosa F, Varoquaux G, Gramfort A, Michel V, Thirion B, Grisel O, et al. Scikit-learn: machine learning in Python. J Mach Learn Res. 2011;12:2825–30.

[29] Seabold S, Perktold J. Statsmodels: Econometric and Statistical Modeling with Python. In: Proceedings of the Python in Science Conference, 2010. 92–6. 10.25080/majora-92bf1922-011

[30] Hunter JD. Matplotlib: A 2D graphics environment. Comput Sci Eng. 2007;9: 90–5. doi: 10.1109/MCSE.2007.55

[31] Waskom M. seaborn: statistical data visualization. JOSS. 2021;6(60):3021. doi: 10.21105/joss.03021

[32] Hirooka K, Shioda S, Okada M. Identification of critical residues for the catalytic activity of ComQ, a Bacillus prenylation enzyme for quorum sensing, by using a simple bioassay system. Biosci Biotechnol Biochem. 2020;84(2):347–57. doi: 10.1080/09168451.2019.1685371 31670609

[33] Kada S, Ishikawa A, Ohshima Y, Yoshida K. Alkaline serine protease AprE plays an essential role in poly-γ-glutamate production during natto fermentation. Biosci Biotechnol Biochem. 2013;77(4):802–9. doi: 10.1271/bbb.120965 23563567

[34] Kimura K, Itoh Y. Determination and characterization of IS4Bsu1-insertion loci and identification of a new insertion sequence element of the IS256 family in a natto starter. Biosci Biotechnol Biochem. 2007;71(10):2458–64. doi: 10.1271/bbb.70223 17928718

[35] Špacapan M, Danevčič T, Štefanic P, Porter M, Stanley-Wall NR, Mandic-Mulec I. The ComX Quorum Sensing Peptide of Bacillus subtilis Affects Biofilm Formation Negatively and Sporulation Positively. Microorganisms. 2020;8(8):1131. doi: 10.3390/microorganisms8081131 32727033 PMC7463575

[36] Rooney AP, Price NPJ, Ehrhardt C, Swezey JL, Bannan JD. Phylogeny and molecular taxonomy of the Bacillus subtilis species complex and description of Bacillus subtilis subsp. inaquosorum subsp. nov. Int J Syst Evol Microbiol. 2009;59(Pt 10):2429–36. doi: 10.1099/ijs.0.009126-0 19622642

[37] Dobrzyński J, Wróbel B, Górska EB. Taxonomy, Ecology, and Cellulolytic Properties of the Genus Bacillus and Related Genera. Agriculture. 2023;13(10):1979. doi: 10.3390/agriculture13101979

[38] Tran LS, Nagai T, Itoh Y. Divergent structure of the ComQXPA quorum-sensing components: molecular basis of strain-specific communication mechanism in Bacillus subtilis. Mol Microbiol. 2000;37(5):1159–71. doi: 10.1046/j.1365-2958.2000.02069.x 10972833

[39] Bustin SA, Benes V, Garson JA, Hellemans J, Huggett J, Kubista M, et al. The MIQE guidelines: minimum information for publication of quantitative real-time PCR experiments. Clin Chem. 2009;55(4):611–22. doi: 10.1373/clinchem.2008.112797 19246619

[40] Tajadini M, Panjehpour M, Javanmard SH. Comparison of SYBR Green and TaqMan methods in quantitative real-time polymerase chain reaction analysis of four adenosine receptor subtypes. Adv Biomed Res. 2014;3:85. doi: 10.4103/2277-9175.127998 24761393 PMC3988599

[41] Nagai T, Tran LS, Inatsu Y, Itoh Y. A new IS4 family insertion sequence, IS4Bsu1, responsible for genetic instability of poly-gamma-glutamic acid production in Bacillus subtilis. J Bacteriol. 2000;182(9):2387–92. doi: 10.1128/JB.182.9.2387-2392.2000 10762236 PMC111298

