# Supplementary figures and images for: Development of a rapid comQ-targeted quantitative polymerase chain reaction assay for specific identification and quantification of Bacillus subtilis subsp. natto
Source: PLoS One. 2026 Aug 3;21(8):e0355394. doi: 10.1371/journal.pone.0355394 (PMC13432125; doi:10.1371/journal.pone.0355394)

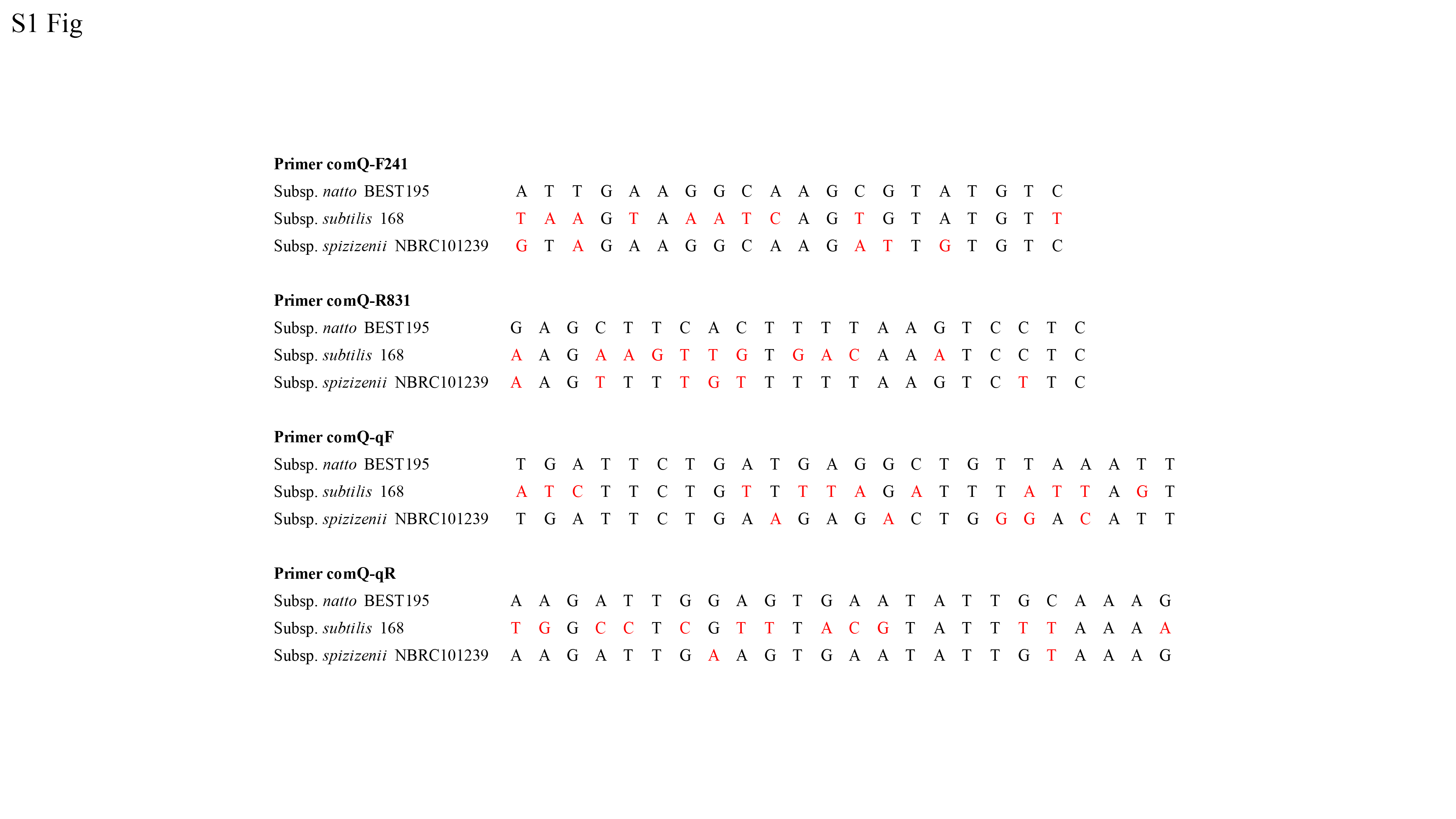

Supplement: S1 Fig — Multiple sequence alignment of the comQ gene from B. subtilis subsp. natto BEST195, B. subtilis subsp. subtilis 168, and B. subtilis subsp. subtilis NBRC 101239. Red letters indicate mismatched nucleotides relative to the BEST195 sequence. Arrows indicate the primer binding sites and directions. (TIF) [file pone.0355394.s005.tif]

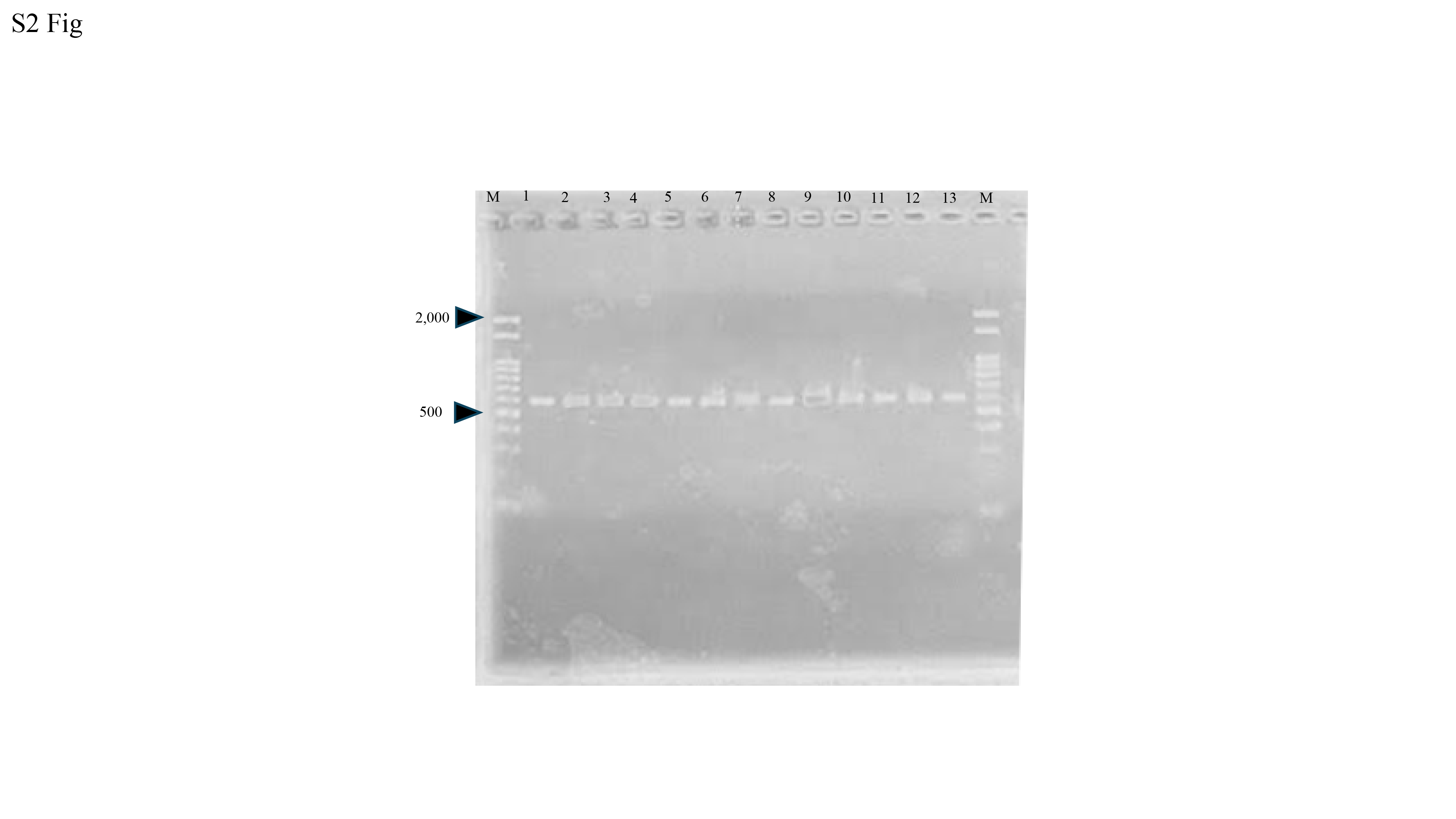

Supplement: S2 Fig — PCR products were amplified using the primer set comQ-241F and comQ-831R and analyzed via agarose gel electrophoresis. Lanes 1–13, NBRC 3013, Miyagino, Takahashi, Naruse, HK1−1, HK11−1, HK3−1, NBRC 3336, NBRC 16449, NBRC 3009, NBRC 3335, NBRC 3936, and NBRC 13169. Lane M, Gene-Ladder 100 (Nippon Gene Co., Ltd., Tokyo, Japan); black triangles indicate 2,000 and 500 bp. Gel images were converted to grayscale and adjusted for contrast using Adobe Photoshop 2020. The images shown are representative results from three independent experiments. (TIF) [file pone.0355394.s006.tif]

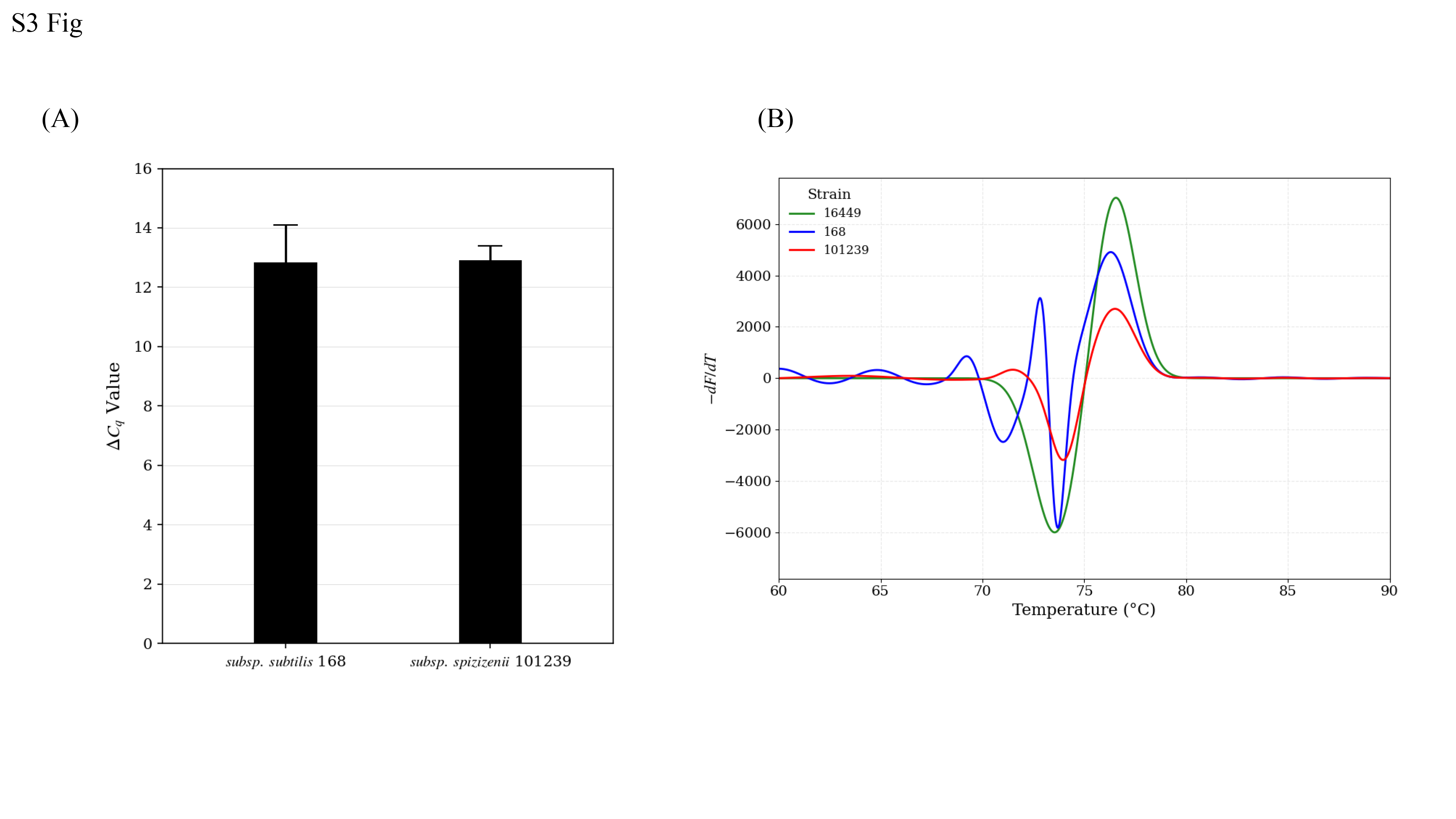

Supplement: S3 Fig — (A) Difference in Cq values (ΔCq) between the target and nontarget strains. (B) Derivative melting curve analysis (−dF/dT) showing the distinct melting peaks for the target and nontarget strains. The green, blue, and red lines represent the subsp. natto-specific target (NBRC 16449), B. subtilis 168, and B. subtilis NBRC 101239, respectively. Both (A) and (B) were generated from the mean values from three independent experiments, with the exception of the data for strain NBRC 16449, which were based on five independent experiments. (TIF) [file pone.0355394.s007.tif]

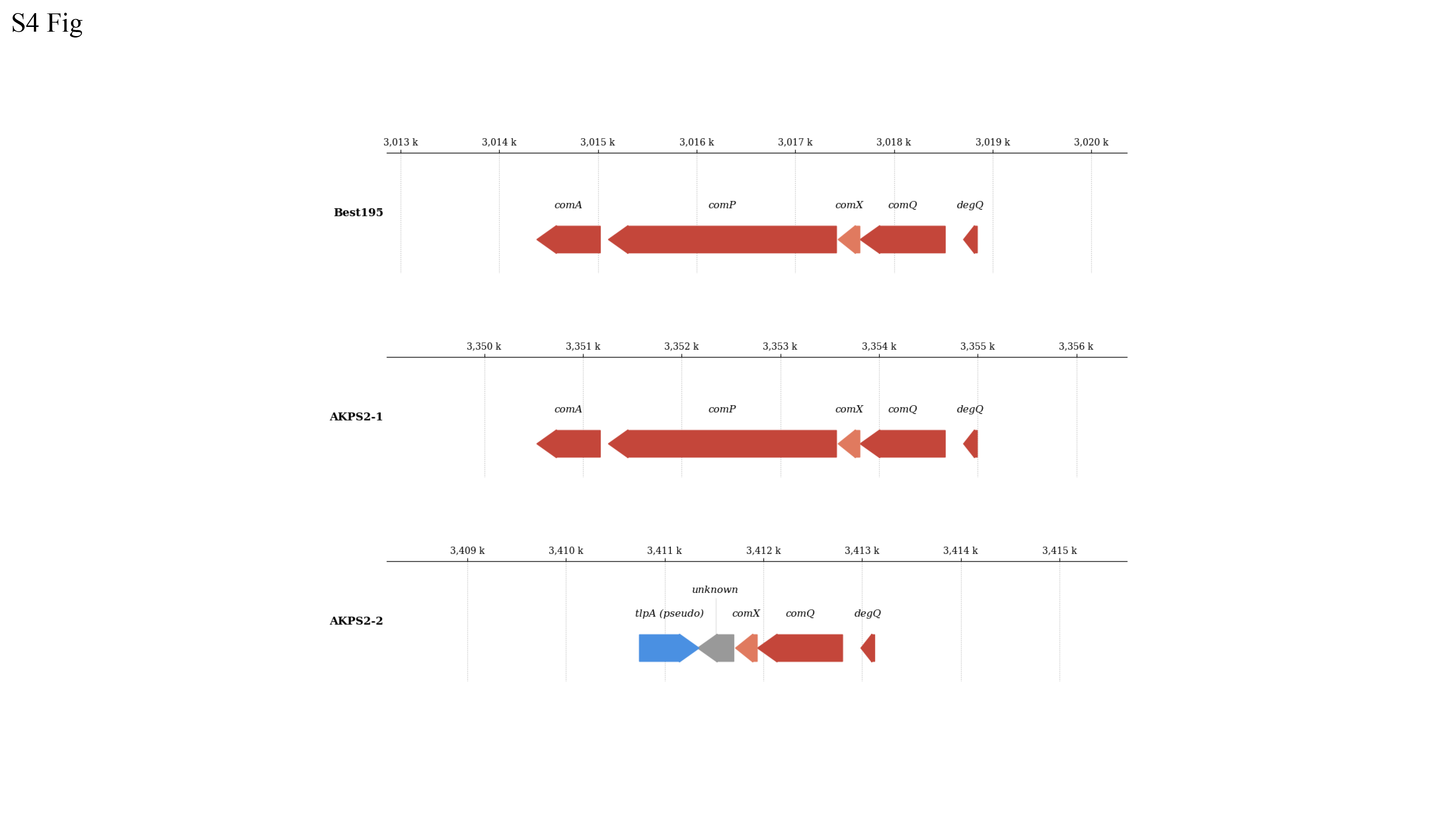

Supplement: S4 Fig — Comparative schematic representation of the com gene clusters among the reference strain BEST195 and the two duplicated loci in strain AKPS2 (designated as AKPS2−1 and AKPS2−2). Arrows indicate the position, length, and transcriptional direction of each gene. Red arrows represent the core quorum-sensing components (comA, comP, comX, comQ) and degQ. Blue and gray arrows indicate the tlpA pseudogene and the uncharacterized gene, respectively. Genomic coordinates (kb) are indicated above the horizontal line for each locus. (TIF) [file pone.0355394.s008.tif]

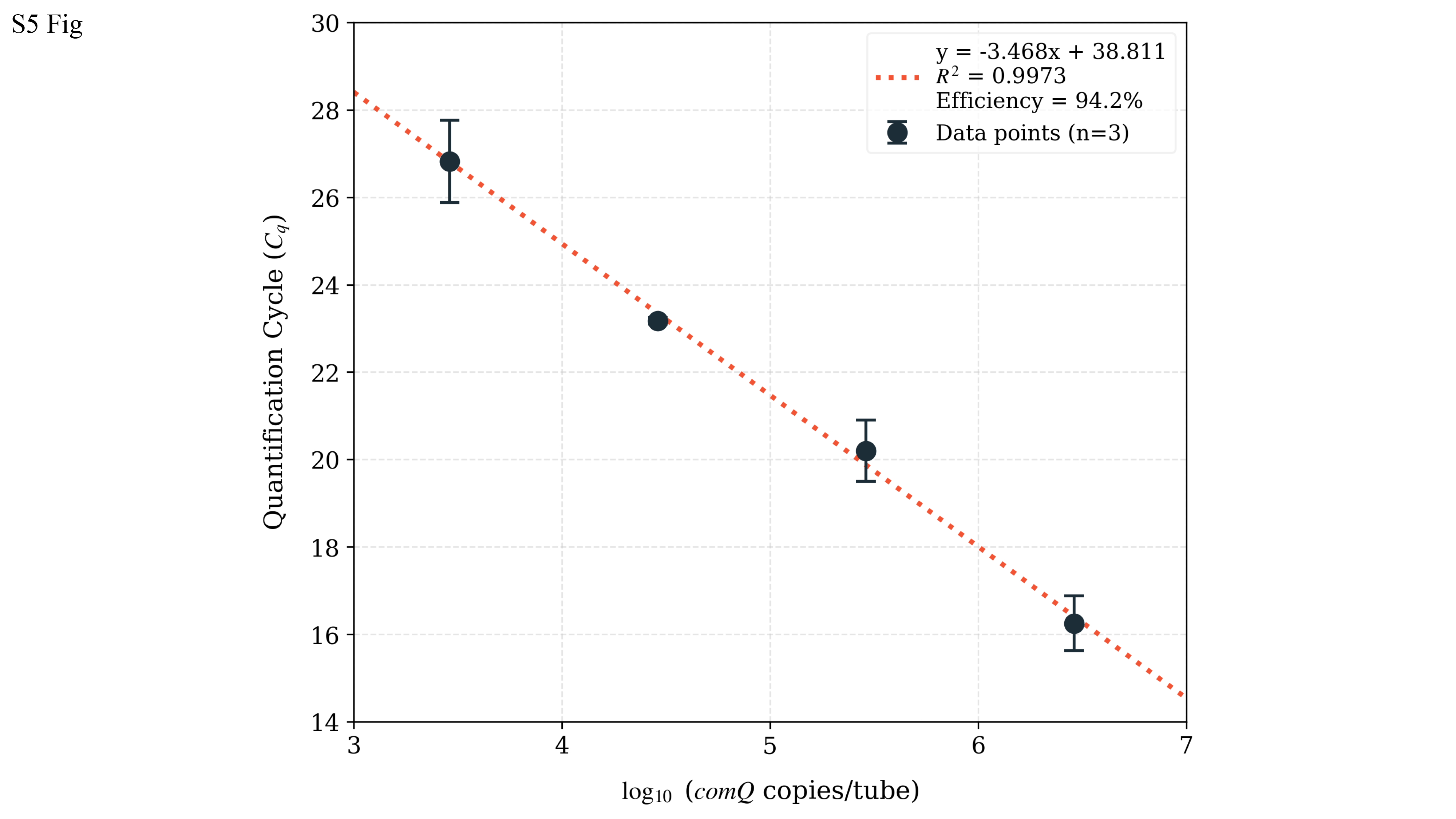

Supplement: S5 Fig — The quantification cycle (Cq) values are plotted against the log10-transformed copy numbers of the comQ gene fragment per reaction tube. The dotted red line represents the linear regression model derived from the experimental data. Each data point represents the mean value obtained from three independent replicates (n = 3), with error bars indicating the standard deviation (SD). (TIF) [file pone.0355394.s009.tif]
